# *Mycobacterium tuberculosis* H37Ra: a surrogate for the expression of conserved, multimeric proteins of *M.tb* H37Rv

**DOI:** 10.1186/s12934-016-0537-0

**Published:** 2016-08-11

**Authors:** Vishant Mahendra Boradia, Pravinkumar Patil, Anushri Agnihotri, Ajay Kumar, Kalpesh Kumar Rajwadi, Ankit Sahu, Naveen Bhagath, Navdeep Sheokand, Manoj Kumar, Himanshu Malhotra, Rachita Patkar, Navi Hasan, Manoj Raje, Chaaya Iyengar Raje

**Affiliations:** 1 National Institute of Pharmaceutical Education and Research (NIPER), Phase X, Sector 67, SAS Nagar, Punjab 160062 India; 2 Council of Scientific and Industrial Research-Institute of Microbial Technology (CSIR-IMTECH), Sector 39 A, Chandigarh, 160036 India; 3 Department of Biotechnology, National Institute of Pharmaceutical Education and Research (NIPER), Phase X, SAS Nagar, Punjab 160062 India

## Abstract

**Background:**

Obtaining sufficient quantities of recombinant *M.tb* proteins using traditional approaches is often unsuccessful. Several enzymes of the glycolytic cycle are known to be multifunctional, however relatively few enzymes from *M.tb* H37Rv have been characterized in the context of their enzymatic and pleiotropic roles. One of the primary reasons is the difficulty in obtaining sufficient amounts of functionally active protein.

**Results:**

In the current study, using *M.tb* glyceraldehyde-3-phosphate dehydrogenase (GAPDH) we demonstrate that expression in *E. coli* or *M. smegmatis* results in insolubility and improper subcellular localization. In addition, expression of such conserved multisubunit proteins poses the problem of heteromerization with host homologues. Importantly the expression host dramatically affected the yield and functionality of GAPDH in terms of both enzymatic activity and moonlighting function (transferrin binding). The applicability of this system was further confirmed using two additional enzymes i.e. *M.tb* Pyruvate kinase and Enolase.

**Conclusions:**

Our studies establish that the attenuated strain *M.tb* H37Ra is a suitable host for the expression of highly hydrophobic, conserved, multimeric proteins of *M.tb* H37Rv. Significantly, this expression host overcomes the limitations of *E. coli* and *M. smegmatis* expression and yields recombinant protein that is qualitatively superior to that obtained by traditional methods. The current study highlights the fact that protein functionality (which is an an essential requirement for all in vitro assays and drug development) may be altered by the choice of expression host.

**Electronic supplementary material:**

The online version of this article (doi:10.1186/s12934-016-0537-0) contains supplementary material, which is available to authorized users.

## Background

Globally several laboratories are involved in identifying *Mycobacterium tuberculosis* (*M.tb*) proteins that play a crucial role in immunomuodulation, gene regulation, pathogenesis and virulence with the aim of developing novel anti-mycobacterials. Functional characterization of these activities requires large quantities of purified protein and commonly, recombinant *M.tb* proteins are expressed in available *E. coli* strains. However, due to their high GC content and intrinsic hydrophobicity the products are often functionally inactive. To overcome this problem multiple approaches have been attempted including: (1) optimization of refolding or growth conditions [1], (2) changing the tag or strain to enhance solubility [2], (3) creating strains with compatible codon usage [3] and (4) expression in *M. smegmatis* which is a fast growing strain, closely related to *M. tuberculosis* [4–6].

Numerous microbial pathogens utilize the alternate functions of glycolytic and TCA cycle enzymes to enhance their virulence [7]. Bacterial GAPDH has been associated with multiple functions including as an adhesin, invasin and an EGF binding protein [7, 8]. Recently we demonstrated that in pathogenic *M.tb* H37Rv, GAPDH and other conserved proteins function as receptors for the uptake of transferrin and consequent iron acquisition by the pathogen [9].

As reported previously, *M.tb* GAPDH has significant solubility issues and complete characterization of the enzyme has not been possible [10]. While attempting to validate the role of *M.tb* GAPDH as a transferrin receptor we were unable to obtain sufficient quantities of stable recombinant protein using *E. coli* [10] or *M. smegmatis* as host strains.

The current study demonstrates that *M.tb* H37Ra can be utilized as a host for the successful expression and purification of recombinant *M.tb* H37Rv proteins. Expression in *M. tuberculosis* H37Ra exhibited ~99 % purity with an improved yield of stable and functionally active enzyme. The majority of recombinant protein was localized in the cytosol as opposed to significant incorporation into the membrane and cell wall fractions upon expression in *E. coli* or *M. smegmatis*. In addition as GAPDH is a highly conserved multisubunit protein we also observed that expression in *M. smegmatis* resulted in the formation of a chimeric protein incorporating both host and vector components. Functional characterization revealed that enzyme activity significantly differed between proteins purified from each of these hosts. The alternate moonlighting function of GAPDH i.e. transferrin binding also varied depending on the source of the protein. To confirm the utility of this expression system, two additional multimeric *M.tb* proteins Enolase and Pyruvate kinase (PykA) were also successfully expressed in *M.tb* H37Ra. As with GAPDH, we observed significant differences in the yield, heteromerization and enzyme activity as compared to recombinant protein obtained from *E. coli or M. smegmatis* host strains. Overall, this study identifies an expression system suitable for *M.tb* H37Rv proteins that retains both solubility and functionality. This study encompasses aspects of tuberculosis research that require recombinant proteins of the human pathogenic strain *M.tb* H37Rv including enzymology, host-pathogen interaction or screening for vaccine and drug candidates.

## Results

### Subcellular localization of rGAPDH

Recombinant *M.tb* GAPDH (rGAPDH) was expressed in three different strains viz*. E. coli*, *M. smegmatis* and *M. tuberculosis* H37Ra, protein purified from these host strains are referred to as EC-H-GAPDH, MS-H-GAPDH, MT-H-GAPDH, MS-GAPDH-H, MT-GAPDH-H to reflect the expression host, position of Histidine tag and protein.

**Fig. 1:**
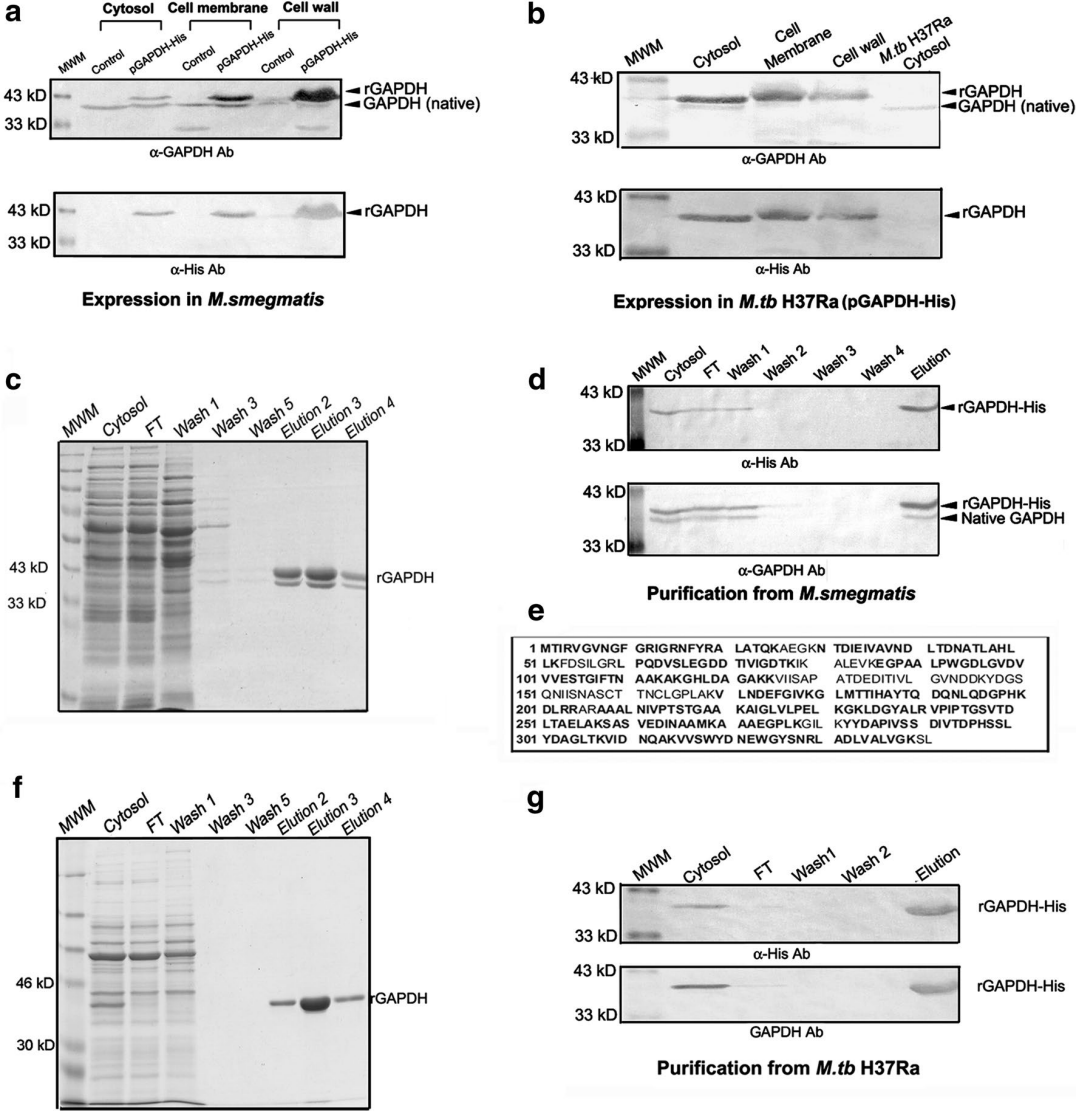
Localization and purification of recombinant *M.tb* GAPDH. **a** Localization of rGAPDH in cellular fractions of *M. smegmatis* detection was done using α-GAPDH and α-His antibodies. **b** Localization of rGAPDH in cellular fractions of *M.tb H37Ra* detection was done using α-GAPDH and α-His antibodies. **c** Affinity purification of MS-GAPDH-H, analysis by 10 % SDS-PAGE. **d** Western blot with α-GAPDH and α-His to confirm purification from *M. smegmatis*. **e** Peptide mass fingerprinting (PMF) of 40 kD *M. smegmatis* protein that co-elutes with rGAPDH. Identified sequences are indicated in *bold face*. **f** Affinity purification of MT-GAPDH-H. **g** Western blot with α-GAPDH and α-His to confirm purification from *M. tb* H37Ra

Localization of rGAPDH was evaluated in different cell fractions of *E. coli BL21DE3* GroEL/GroES. A majority of protein was present in the cell wall and inclusion body fractions (Additional file 1: Figure 1a, b). The GAPDH gene was cloned in a high copy number *E. coli*-*M.tb* shuttle vector to allow expression of the His-tagged protein in *M. smegmatis*. Cytosol, cell membrane and cell wall fractions were prepared from control (untransformed) or rGAPDH expressing *M. smegmatis* strains. The presence of GAPDH was confirmed with α-GAPDH (which detects GAPDH from *M.tb* as well as *M. smegmatis*) [9] and α-His antibodies to confirm the presence of rGAPDH-His (Fig. 1a; Additional file 3: Table 1). A single band of 40 kD corresponding to the expected size of the native *M. smegmatis* GAPDH was detected in all fractions of the untransformed strain (Fig. 1a). In transformed cells, besides native GAPDH, an additional 42 kD protein corresponding to the expected size of rGAPDH was observed. Though rGAPDH was present in all fractions it was predominantly localized in the cell wall (Fig. 1a). Reprobing with α-His antibody confirmed that the 42 kD protein was rGAPDH (Fig. 1a).

Next, *M.tb* H37Ra was transformed with the same construct, western blots probed with α-GAPDH and α-His antibodies (Fig. 1b) confirmed expression of the 42 kD rGAPDH in all fractions, in accordance with previous results where parent strains of both *M.tb H37Ra* and *M.tb H37Rv* demonstrate the presence of GAPDH in all subcellular fractions [9, 11–13]. In controls (untransformed) the 40 kD native GAPDH was detected with α-GAPDH antibody. As compared to the expression observed in *M. smegmatis*, only a minor amount of native GAPDH was detected in the cytosol of the *M.tb* H37Ra transformant, indicating that rGAPDH is highly expressed and constitutes the predominant species (Fig. 1a, b).

### Protein purification by Ni–NTA chromatography

Purification of EC-H-GAPDH from the soluble fraction was done using published protocols [10] and confirmed by SDS-PAGE and western blotting (Additional file 1: Figure 1c). Unlike previous reports on purification from *E. coli* BL21DE3 [10], we found that supplementation with 1.0 mM NAD^+^ was not essential during purification from either mycobacterial host. However, affinity purified MS-GAPDH-H demonstrated the presence of two distinct proteins of ~42 kD (rGAPDH) and ~40 kD (native *M. smegmatis* GAPDH) (Fig. 1c). Detection with α-His antibody, confirmed that only the ~42 kD protein was rGAPDH however both proteins were detectable with α-GAPDH (Fig. 1d). The ~40 kD protein band was identified to be that of native *M. smegmatis* GAPDH (79 % coverage, MOWSE score 44) by peptide mass fingerprinting (Fig. 1e). The observation that native *M. smegmatis* GAPDH co-purifies with MS-GAPDH-H suggests the formation of heteromers. In contrast, purification from *M.tb* H37Ra resulted in a single prominent band of ~42 kD of rGAPDH (Fig. 1f), as confirmed by western blotting with both antibodies (Fig. 1g).

Intact mass analysis of purified recombinant protein from *M.tb* H37Ra and *E. coli* revealed the presence of a single predominant species of 37.99 and 38.144 kD respectively (Additional file 2: Figure 2a, b, Additional file 3: Table 2). Purified recombinant proteins from all three sources were then subjected to 2D gel electrophoresis (Additional file 2: Figure 2c–e). Trace amounts of associated native GAPDH was detected upon expression in *M.tb* H37Ra (pI 5.6) and *M. smegmatis* (pI 5.42) hosts reconfirming the formation of heteromers (Additional file 2: Figure 2c, d). The pI of recombinant MT-GAPDH-H, MS-GAPDH-H and EC-H GAPDH were 6.43, 6.37 and 6.57 respectively (Additional file 2: Figure 2c–e, Additional file 3: Table 3). As expected, GAPDH sequences from *M.tb* H37Rv and *M.tb* H37Ra strains were identical. *M. smegmatis* GAPDH demonstrates a high degree of sequence identity (88 % identity and 94 % similarity) which could account for heteromer formation with rGAPDH. *E. coli* GAPDH shares 52 % identity and 69 % similarity with *M.tb* H37Rv GAPDH (Additional file 1: Figure 1d).

**Fig. 2:**
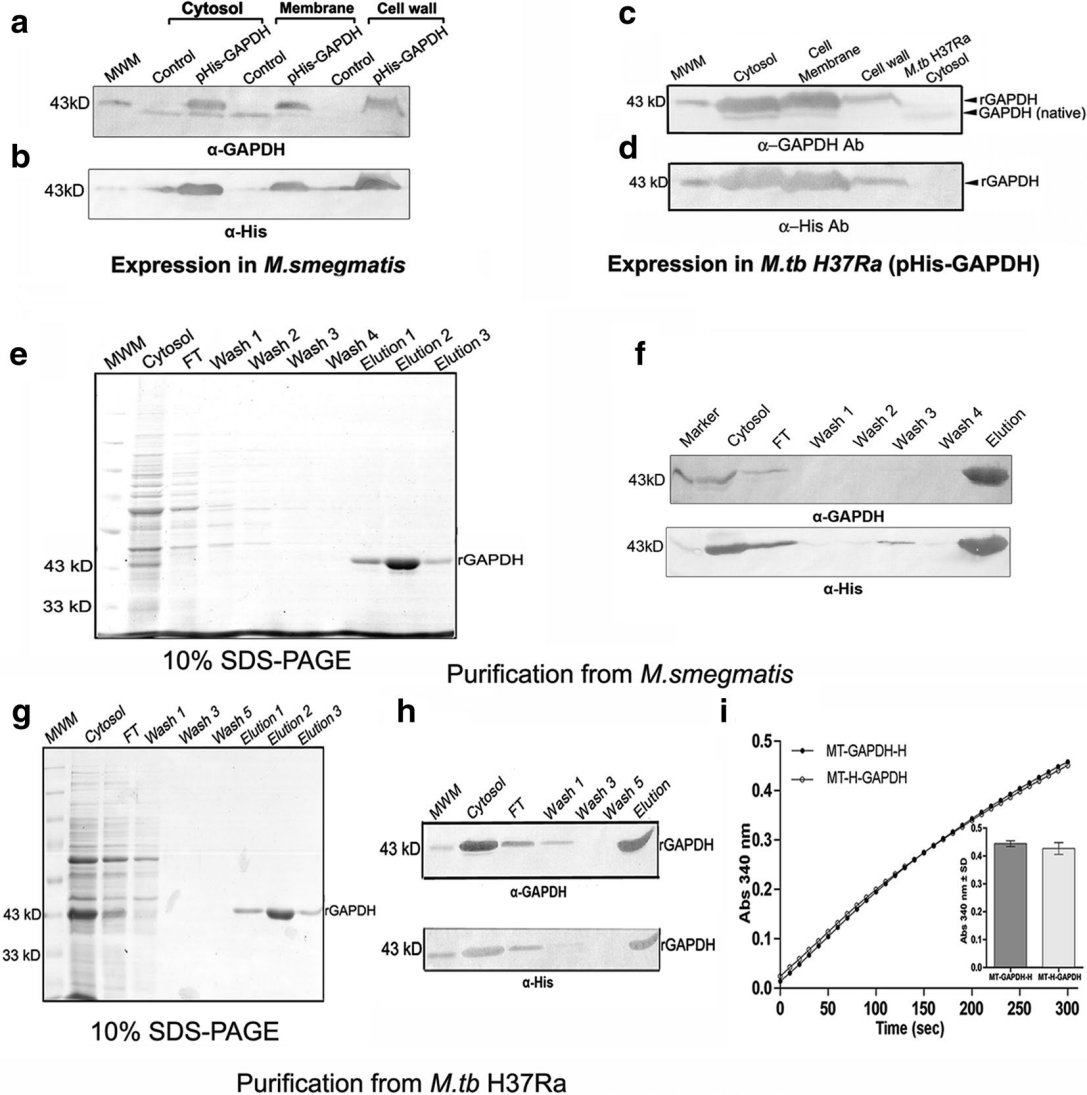
Localization and purification of recombinant N-terminal His tagged *M.tb* GAPDH from *M. smegmatis* and *M.tb* H37Ra strains: Localization of rH-GAPDH in cellular fractions of *M. smegmatis* detection was done using **a** α-GAPDH and **b** α-His antibodies. Localization of rGAPDH in cellular fractions of *M.tb H37Ra* detection was done using **c** α-GAPDH and **d** α-His antibodies. **e** Affinity purification of MS-H-GAPDH, analysis by 10 % SDS-PAGE. **f** Western blot with α-GAPDH and α-His to confirm purification from *M. smegmatis*. **g** Affinity purification of MT-H-GAPDH. **h** Western blot with α-GAPDH and α-His to confirm purification from *M. tb* H37Ra. **i** Comparative enzyme activity of N- (MT-H-GAPDH) and C-terminal (MT-GAPDH-H) His tagged recombinant GAPDH purified from *M.tb* H37Ra. Inset: Comparison of end point values, experiment was repeated three times (n = 3), data is represented as absorbance at 340 nm ±SD

Since the experimental and theoretical intact mass values differed, the post translational modifications (PTMs) of rGAPDH purified from individual hosts were evaluated by LC–MS/MS. PTM’s in *M.tb* influence various biological activities such as protease resistance, stability, compartmentalization, membrane anchoring, protein–ligand interactions and protein degradation [14]. Analysis revealed that MT-GAPDH-H had enhanced PTMs, including phosphorylation, deamidation, methylation and succinylation as compared to EC-H-GAPDH. The highest number of PTMs (69) were observed in MS-GAPDH-H, in comparison native *M. smegmatis* GAPDH had far fewer modifications (36). Phosphorylation, deamidation, methylation, dimethylation, pro-pyro-glu and acetylation were all enhanced in MS-GAPDH-H as compared to native *M. smegmatis* GAPDH (Additional file 3: Table 4). Detailed methodology for Peptide mass fingerprinting, 2D gel electrophoresis, sequence analysis and LC MS/MS are provided (Additional file 4). 

### Expression and purification of MS-H-GAPDH and MT-H-GAPDH

**Fig. 3:**
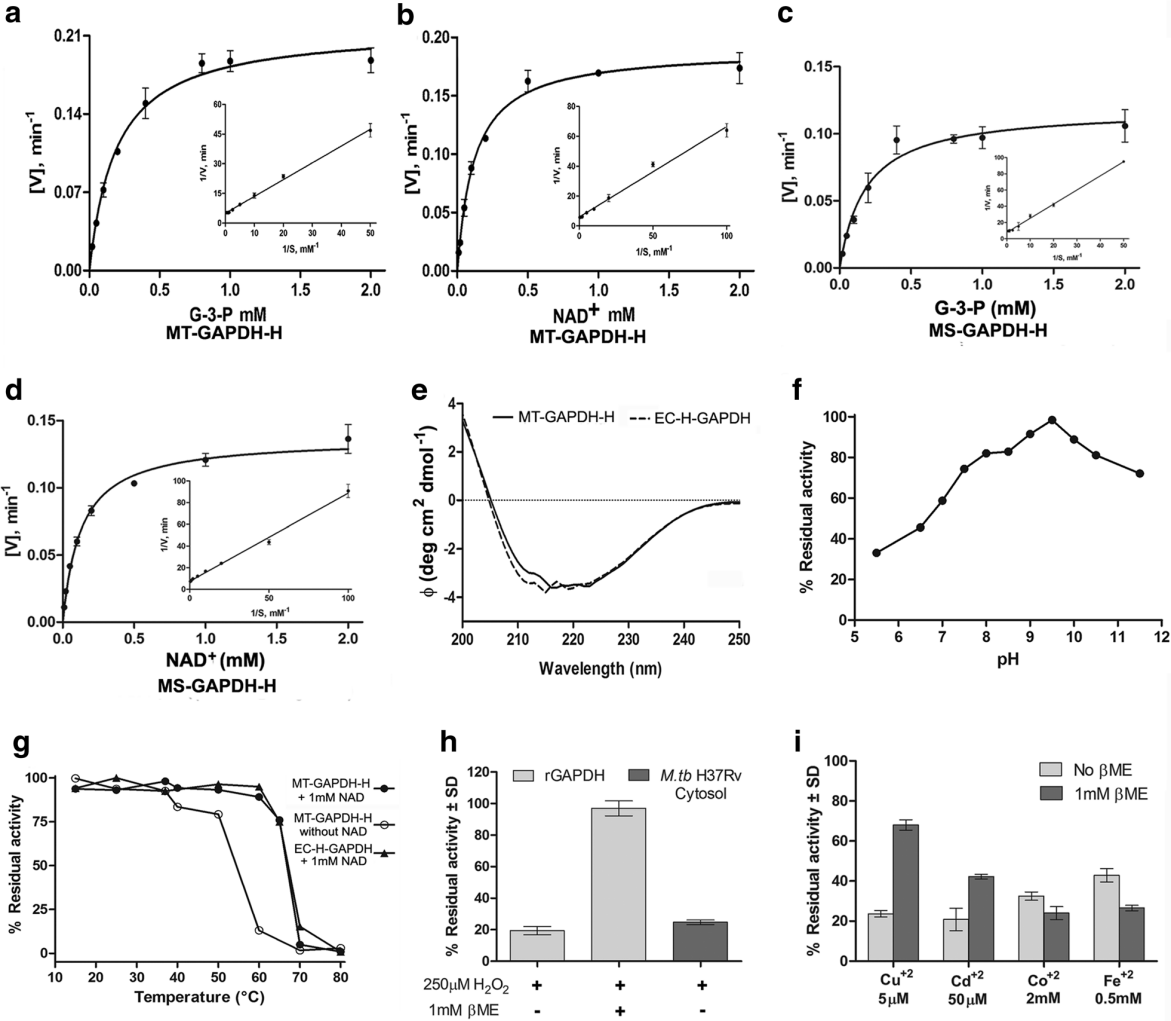
Characterization of rGAPDH: determination of K_m_ and V_max_ for G3P and NAD^+^ of **a**, **b** MT-GAPDH-H and **c**, **d** MS-GAPDH-H. The extent of enzymatic conversion was monitored by the formation of NADH at 340 nm at 25 °C. **a**, **c** The substrate NAD^+^ was added in excess (1 mM) while G3P was varied (0.02–2 mM). **b**, **d** Substrate G3P was in excess (2 mM), while NAD^+^ was varied from 0.01 to 2 mM. Data is fitted to Michaelis–Menten equation. *Inset* Data represented as a Lineweaver–Burk plot. **e** Comparative CD spectra of rGAPDH purified of MT-GAPDH-H and EC-H-GAPDH. **f** The activity was measured for 2 min, data is plotted as % residual activity versus pH. Average of three independent experiments is represented. **g** Temperature stability of rGAPDH-His. Data is plotted as % residual activity representing the average of three independent experiments. **h** Sensitivity of MT-GAPDH-H and *M.tb* H37Rv cytosol to H_2_O_2_ in the presence or absence of β-ME. **i** Sensitivity of MT-GAPDH-H to metal ions in the presence or absence of β-ME. Data is represented as % residual activity ±SD, n = 3

Expression of N-terminal tagged His tagged protein was also carried out in *M. smegmatis* (Fig. 2a, b) and *M.tb* H37Ra (Fig. 3c, d). The fractionation results were comparable to expression of C-terminal tagged protein. Protein was purified from *M. smegmatis* (Fig. 2e, f) and *M.tb* H37Ra (Fig. 2g, h). The enzyme activity of recombinant N-and C-His tagged GAPDH purified from *M.tb* H37Ra was comparable, indicating that position of the His tag did not affect enzyme activity (Fig. 2i).

### Enzyme kinetics

A recent study on the purification and characterization of rGAPDH from *E. coli*, indicated that the enzyme is unstable in the absence of NAD^+^ [10]. In contrast to those observations, our investigations revealed that NAD^+^ supplementation was not necessary for purification of protein expressed in either mycobacterial host strain. The kinetic parameters for MT-GAPDH-H and MS-GAPDH-H were evaluated to ascertain whether they are at variance with reported values for *E. coli* expressed enzyme [10].

The K_m_ and V_max_ (G3P) of MT-GAPDH-H (Fig. 3a) and the corresponding values for MS-GAPDH-H were estimated (Fig. 3c). However, the reported values (K_m_ 280 ± 30 µM and V_max_ 1670 ± 90 min^−1^) [10] for *M.tb* rGAPDH purified from *E. coli* are very different. Similarly, the K_m_ (NAD^+^) of recombinant enzyme for MT-GAPDH-H (Fig. 3b) and of MS-GAPDH-H (Fig. 3d) were different from the K_m_ of 40 ± 4 µM reported previously for *M.tb* H37Rv rGAPDH purified from *E. coli*, perhaps because the enzyme used for these experiments was saturated with NAD^+^ during the purification process. [10]. Data is summarized in Table 1.

**Table 1:** K_m_ and V_max_ values of rGAPDH purified from different host strains

Host	K_m_ (G3P) (µM)	V_max_ (G3P) (min^−1^)	K_m_ (NAD^+^) (µM)
*E. coli* BL21DE3 ^13^	280 ± 30	1670 ± 90	
	40 ± 4		
*M. smegmatis*	223.8 ± 4.66	0.128 ± 0.0023	109.4 ± 3.4
*M.tb* H37Ra	169.6 ± 10.53	0.199 ± 0.012	101.2 ± 8.9

### Circular dichroism (CD spectroscopy)

Since K_m_ and V_max_ values for rGAPDH varied considerably we considered whether these differences could be attributed to structural differences. The CD spectra of EC-H GAPDH and MT-GAPDH-H were determined and it was established that secondary structures are essentially comparable with an α-helicity of 28 % (Fig. 3e).

### pH stability

In contrast to previous findings that *E. coli* derived rGAPDH was unstable outside the pH range of 5.5–8.5 [10], MT-GAPDH-H retained 70 % residual activity even in the pH range of 7.5–11.5, with an optimal stability at pH 9.5, however, below pH 7.5, a significant loss of enzyme activity was observed (Fig. 3f).

### Thermal denaturation

In the presence of 1 mM NAD^+^, both MT-GAPDH-H and EC-H-GAPDH were stable in the temperature range of 15–65 °C. In the absence of 1 mM NAD^+^ a decrease in enzyme activity of MT-GAPDH-H was observed in the range of 37–50 °C, with a drastic loss of enzyme activity at 60 °C. Above 65 °C the enzyme lost activity irrespective of the presence or absence of NAD^+^ (Fig. 3g). EC-H-GAPDH could not be assessed without NAD^+^ due to instability of the enzyme in these conditions.

### Effect of H_2_O_2_ and metal ions

GAPDH from other species is known to be a redox sensitive protein containing an active site cysteine residue which is susceptible to oxidation [15]. Disulfide bond formation results in decreased enzymatic activity, which can be minimized by addition of the reductant 2-mercaptoethanol (β-ME) [10]. In case of MT-GAPDH-H and *M.tb* H37Rv cytosol, only 20 % activity was retained in the presence of 250 µM H_2_O_2_. In contrast enzyme activity was unaffected by 250 µM H_2_O_2_ in the presence of 1 mM β-ME (Fig. 3h). All divalent metal ions tested (Cd^2+^, Cu^2+^, Co^2+^, Fe^2+^) inhibited enzyme activity to varying extents irrespective of the presence of 1 mM β-ME (Fig. 3i).

### Modulation of multifunctional activity

**Fig. 4:**
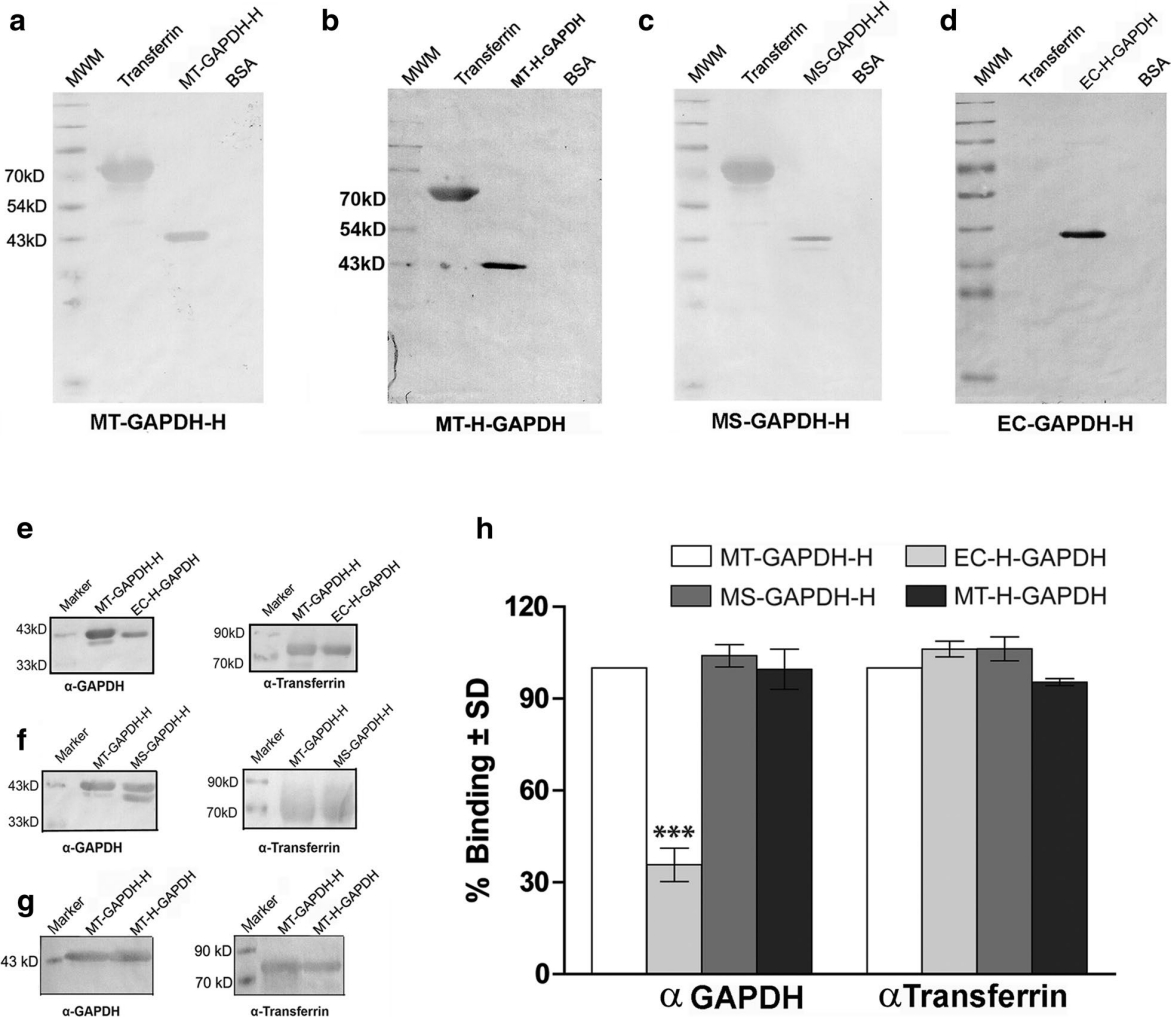
Host strain affects the moonlighting function (transferrin binding) of rGAPDH: far western blotting demonstrates that binding of recombinant GAPDH to transferrin varies with the host strain. **a** MT-GAPDH-H, **b** MT-H-GAPDH, **c** MS-GAPDH-H and **d** EC-H-GAPDH. The interaction of GAPDH with transferrin was detected using polyclonal rabbit α GAPDH antibody. rGAPDH from each source represents the positive control while BSA has been utilized as a negative control. Co-immunoprecipitation analysis comparing capture of: **e** MT-GAPDH-H and EC-H-GAPDH, **f** MT-GAPDH-H and MS-GAPDH-H. **g** MT-GAPDH-H and MT-H-GAPDH Blots were probed with polyclonal rabbit α GAPDH antibody (to determine capture) and α transferrin antibody (to confirm that equal amounts of transferrin were used). **h** Densitometry analysis, co-immunoprecipitation of MT-GAPDH-H was taken as 100 %. Experiments were repeated multiple times, data is represented as a histogram depicting % binding ± SD, n = 3, ***p ≤ 0.001

Apart from enzymatic properties we evaluated whether moonlighting functions were also affected. Far western blotting was utilized to confirm the interaction of transferrin with rGAPDH individually purified from each host. While MT-GAPDH-H, MT–H-GAPDH and MS-GAPDH-H demonstrated a distinct interaction with human transferrin (Fig. 4a–c) EC-H-GAPDH failed to capture any detectable levels of transferrin (Fig. 4d). Since native conformations are likely to be essential for optimal ligand–receptor interaction at the cell surface, co-immunoprecipitation assays with all four recombinant proteins were carried out (Fig. 4e–g). Densitometry revealed that as compared to MT-GAPDH-H, a 70 % decrease of rGAPDH-transferrin interaction as evident using EC-H-GAPDH (*p* ≤ 0.001) (Fig. 4e, h). No change in GAPDH-transferrin interaction was observed with MS-GAPDH-H (Fig. 4f, h). Both N- and C-terminal 8XHis tagged GAPDH from *M.tb* H37Ra showed comparable results suggesting that the position of the tag did not affect the transferrin binding property (Fig. 4g, h).

### Expression of *M.tb* H37Rv PykA and Enolase in *M.tb* H37Ra

*M.tb* H37Rv PykA and Enolase recombinant proteins have been named EC-PykA-H, MS-PykA-H, MT-PykA-H, EC-H-Enolase, MS-H-Enolase and MT-H-Enolase to indicate the host strain, tag and protein.

**Fig. 5:**
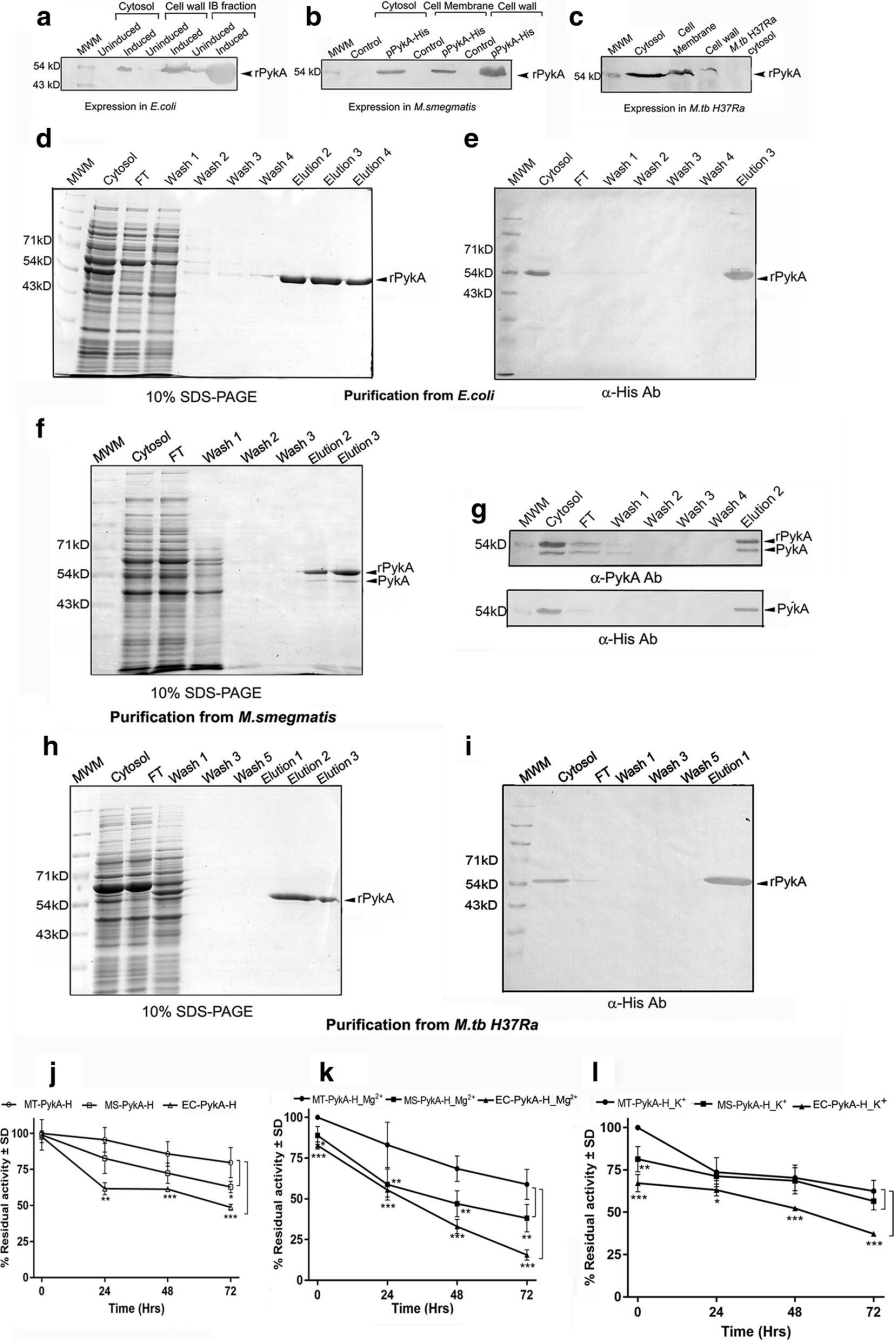
Localization, purification and activity of recombinant *M.tb* PykA in different host strains. **a**–**c** Localization of rPykA-H in cellular fractions of *E. coli* BL21DE3 GroEL/ES strain, *M. smegmatis* and *M.tb* H37Ra respectively, detection was done using α-His antibodies. **d** Affinity purification of EC-PykA-H, analysis by 10 % SDS-PAGE **e** Western blotting with α-His antibody. **f** Affinity purification of MS-PykA-H, analysis by 10 % SDS-PAGE. **g** Western blotting with α-PykA and α-His antibodies. **h** Affinity purification of MT-PykA-H, analysis by 10 % SDS-PAGE. **i** Western blotting with α-His antibodies. Residual enzyme activity (in %) of EC-PykA-H, MS-PykA-H and MT-PykA-H in the **j** absence of cofactors or in the presence of **k** Mg^2+^ or **l** K^+^. Significance was determined by student’s unpaired t-test where *p < 0.05; **p < 0.01 and ***p < 0.001

Expression and purification of *M.tb* PykA was also carried out in all three hosts, in *E. coli*, maximum expression was evident in the inclusion body (IB) fraction (Fig. 5a). In *M. smegmatis* the recombinant protein was detected in all fractions but maximally in the cell wall fraction of (Fig. 5b). In *M.tb* H37Ra while maximum expression was observed in the cytosol, protein was also detected in membrane and cell wall fractions (Fig. 5c). The protein was purified from cytosolic fractions of all three strains (Fig. 5d–i). Upon expression in *M. smegmatis*, heteromer formation with native *M. smegmatis* PykA was evident on SDS-PAGE and western blotting (Fig. 5f, g). The identity of the 50 kD was confirmed to be native *M. smegmatis* PykA by peptide mass fingerprinting, the sequence coverage was 58 % and MOWSE score was 191 (Additional file 2: Figure 2f). In comparison to EC-PykA-H, recombinant MT-PykA-H and MS-PykA-H demonstrated significantly greater stability over time even in the absence of the co-factors Mg^2+^ and K^+^ [16] (Fig. 5j). Supplementation with Mg^2+^ [17] failed to restore the enzyme activity of EC-PykA-H, which was significantly lower than that of MT-PykA-H. MS-PykA-H also demonstrated loss of activity over time as compared to MT-PykA-H (Fig. 5k). Addition of K^+^ marginally improved the stability of EC-PykA-H over time, however both activity and stability were significantly lower than the corresponding values for MT-PykA-H or MS-PykA-H under the same conditions (Fig. 5l).

**Fig. 6:**
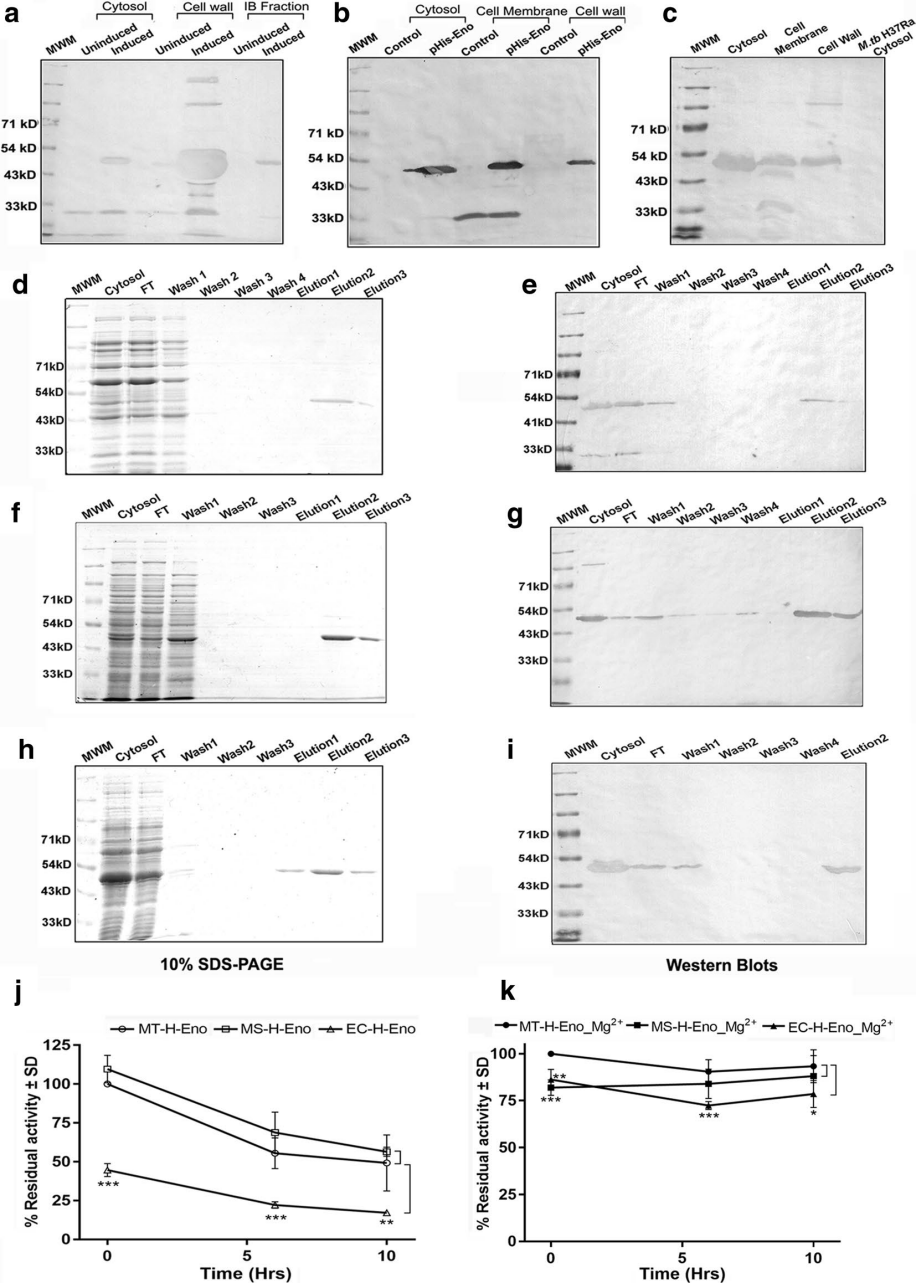
Localization, purification and activity recombinant *M.tb* Enolase in different host strains. **a**–**c** Localization of rH-Enolase in cellular fractions of *E. coli* BL21DE3 GroEL/ES, *M. smegmatis* and *M.tb* H37Ra strains respectively, detection was done using α-His antibodies. **d** Affinity purification of EC-H-Eno, analysis by 10 % SDS-PAGE **e** Western blotting with α-His antibody. **f** Affinity purification of MS-H-Eno), analysis by 10 % SDS-PAGE. **g** Western blotting with α-His antibodies. **h** Affinity purification of MT-H-Eno, analysis by 10 % SDS-PAGE. **i** Western blotting with α-His antibody. Residual enzyme activity (in %) of EC-H-Eno, MS-H-Eno and MT-H-Eno **j** in the absence of Mg^2+^, **k** in the presence of Mg^2+^. Significance was determined by student’s unpaired t test where *p < 0.05; **p < 0.01 and ***p < 0.001

In *E. coli*, EC-H-Eno was maximally localized to the cell wall (Fig. 6a), while in *M. smegmatis* the protein was detected in cytosol, cell membrane and cell wall (Fig. 6b). In *M.tb* H37Ra, maximum expression was detected in the cytosol and to a lesser extent in the cell membrane and cell wall fractions (Fig. 6c). The protein was purified by Ni-affinity chromatography from *E. coli* (Fig. 6d, e) and both mycobacterial strains as confirmed by SDS PAGE and western blotting with anti-His antibody (Fig. 6f–i). All three proteins (EC-H-Eno, MS-H-Eno and MT-H-Eno) were functional, however Mg^2+^ was required to ensure optimal activity (Fig. 6j, k). It was observed EC-H-Eno demonstrated significantly lower activity compared to proteins purified from mycobacterial strains. Supplementation with Mg^2+^ could not fully restore the enzyme activity of EC-H-Eno as compared to MS-H-Eno or MT-H-Eno (Fig. 6j, k).

## Discussion

The current study establishes *M.tb* H37Ra as a suitable alternate host for expression of GAPDH and other proteins from the pathogenic strain *M.tb* H37Rv. The availability of adequate quantities of any purified recombinant protein is essential for definitive in vitro and in vivo studies. *E. coli* has served as a well established expression host for many proteins due the availability of a large number of strains, plasmids and short generation time. However, successful expression of *M.tb* proteins has often been difficult in *E. coli* with major constraints being insolubility and poor yields [18]. To improve the yield of functional protein several strategies such as usage of codon optimized strains, culture conditions and refolding protocols have been considered [1]. As an alternative the fast growing *M. smegmatis* strain has often been as used as a host for expression of *M.tb* proteins [4–6].

While trying to define the alternate roles of the conserved tetrameric glycolytic enzyme GAPDH we encountered difficulties in obtaining sufficient quantities of functional protein. Initially we attempted purification using the expression strains *E. coli* BL21DE3-GroEL/GroES [10] and *M. smegmatis*. In both cases, the yield of rGAPDH was ~1.0 mg/l of culture. As reported previously, supplementation of all buffers with 1 mM NAD^+^ was essential to obtain functionally active rGAPDH from *E. coli* [10]. rGAPDH expressed in *M. smegmatis* was more stable and did not have an absolute requirement for 1 mM NAD^+^ during purification steps. However, we observed that an additional ~40 kD molecule co-elutes along with rGAPDH on denaturing SDS-PAGE. Peptide mass fingerprinting confirmed this to be native *M. smegmatis* GAPDH. The formation of GAPDH hetero-tetramers has been reported previously in other species [19]. It therefore appears that *M. smegmatis* may not be an ideal expression system due to the formation of chimeric proteins. To obtain pure rGAPDH we successfully utilized the attenuated *M.tb* H37Ra strain as an expression system. Intact mass analysis of the recombinant protein (MT-GAPDH-H) was of 37.99 kD which closely corresponds to the theoretical value (37.78 kD). On SDS-PAGE the protein is visible at 42kD, its anomalous mobility could be attributed to its hydrophobicity [20].

Expression in this strain did not require additional supplementation of NAD^+^ during the purification steps. The yield increased to more than twofold (2.5 mg/l of culture), with the prominent species being that of rGAPDH. Since GAPDH has 100 % sequence conservation between *M.tb* H37Ra and *M.tb* H37Rv the presence of minute amounts of contaminating native protein would not significantly affect the end product.

We then carried out biochemical characterization of rGAPDH and defined its functional differences in terms of both enzymatic activity as well as an alternate moonlighting function (transferrin binding) using protein purified from all sources. We found a wide variation between the K_m_ and V_max_ values determined for rGAPDH purified from *M.tb* H37Ra as compared with rGAPDH derived from *M. smegmatis* (this study) or as reported previously for protein expressed in *E. coli* [10]. We then analyzed other parameters such as pH stability, thermal denaturation and effect of metal ions that could not be evaluated in earlier studies [10]. In contrast to previous findings [10], rGAPDH from *M.tb* H37Ra was functional even in the pH range of 7.5–11.5 (Fig. 3f). It is well documented that macrophages, drugs and vitamin C generate superoxide, hydrogen peroxide (H_2_O_2_) and hydroxyl radicals as a bactericidal mechanism [21, 22]. Oxidation by H_2_O_2_ inhibits the activity of several proteins including GAPDH and causes translocation of mammalian GAPDH to the nucleus, where it plays a role in DNA repair [23]. GAPDH is also reported to be inhibited by divalent metal ions [24], our current findings establish that *M.tb* GAPDH enzyme activity is inhibited by both H_2_O_2_ and divalent metal ions. Inhibition by Cu^2+^ and Cd^2+^ was limited in the presence of 1 mM β-ME indicating the role of the active site cysteine residues. CD analysis of *E. coli* and *M.tb* derived rGAPDH suggested that the secondary structures of the two proteins is essentially similar and may not contribute towards observed alterations in kinetic properties and protein stability.

Finally, we analyzed whether the source of rGAPDH affects its role as a transferrin receptor, it was established that rGAPDH derived from *E. coli* does not effectively interact with human holo-transferrin. A 70 % loss in transferrin binding was observed as compared to rGAPDH purified from *M.tb* H37Ra. This is the first report to suggest that a receptor-ligand interaction is altered due to the source of the recombinant protein. The position of the His tag at the N or C-terminus did not affect the purification requirements, enzyme activity or alternate function of protein expressed in *M.tb* H37Ra. In the case of heteromeric protein purified from *M. smegmatis* no change in GAPDH-Tf interaction was observed which can be explained by the fact that *M. smegmatis* native GAPDH itself interacts with transferrin [9].

To establish that this host system is suitable for other *M.tb* proteins, we also assessed the expression of *M.tb* H37Rv pyruvate kinase (Rv1617) and Enolase (Rv0687). Both enzymes have not been fully characterized and are multimeric and multifunctional in *M.tb* and other species [7, 25]. As with GAPDH, maximal cytosolic localization was evident in *M.tb* H37Ra. A large amount of protein was present in the inclusion body fraction (PykA) or cell wall (Enolase) during *E. coli* expression. Dimerization with native protein from *M. smegmatis* was evident in the case of PykA. Expression of *M.tb* pyruvate kinase and Enolase in *E. coli* M15 strain is known to result in insoluble IB associated protein [26]. In the current study, an approximately four-fold increase in yield of Enolase was evident upon purification from *M.tb* H37Ra (12 mg/l) as compared to *E. coli* (2.75 mg/l) in the absence of Mg^2+^. Under these conditions the yield from *M. smegmatis* was 4.5 mg/l. Upon supplementation with Mg^2+^ the yield changed to 16 mg/l (MT-H-Eno); 8.5 mg/l (MS-H-Eno) and to 4 mg/l for EC-H-Eno. The activity and stability of both recombinant proteins obtained from *E. coli* was significantly lower as compared to proteins purified from *M.tb* H37Ra. In the case of PykA, supplementation with K^+^, marginally improved the activity and stability of EC-PykA-H. Enolase from *E. coli* host (EC-H-Eno) consistently demonstrated lower activity (with or without Mg^2+^) as compared to recombinant MT-H-Eno. Pyruvate kinase obtained from *M. smegmatis* strain revealed decreased activity as compared to protein purified from *M.tb* H37Ra.

Proteomic analysis has previously revealed that PTMs can generate a diverse variety of protein species from a single translated molecule which provides cells the ability to rapidly switch functions upon specific demand. In prokaryotes, the role of these modifications is still in the process of being elucidated [14]. Apart from phosphorylation, several studies have identified that lysine acetylation and succinylation are vital for the regulation of protein function. Of the several proteins identified a majority belonged to the category of metabolic enzymes [27, 28]. Such modifications are predicted to regulate the activity of glyoxalate pathway enzymes Isocitrate lyase (Icl) and Malate synthase (GlcB) [27]. In *M.tb*, acetylation and succinylation negatively regulate the enzyme, protein tyrosine phosphatase B (PtpB) [29]. Other reports suggest that while modification sites may be partially conserved the number of modifications and site specificity may vary depending on the organism and strain [30, 31]. In the context of *M.tb*, previous studies have shown that the source of recombinant protein may affect the immunogenicity, protein–protein interaction and antibiotic sensitivity due to changes in PTMs [32, 33]. GAPDH from several bacteria are known to possess post-translational modifications, *M.tb* GAPDH is reported to be phosphorylated, pupylated, succinylated and acetylated [29]. The current study demonstrated that rGAPDH from all three hosts are enzymatically active with significant variation in terms of activity. Our results reveal that the pattern of PTMs demonstrated by rGAPDH varies depending on the host expression strain (Additional file 3: Table 4), and could indicate a role for host specific PTMs in protein function. Detailed studies to elaborate the significance of individual PTMs would be required to fully understand their relevance in protein function.

The broader implications of our studies are that the expression host may drastically affect not only solubility, stability and localization but more importantly functionality of recombinant *M.tb* proteins. Thus the source of a recombinant protein is an important parameter for the outcome of in vitro, in vivo studies such as enzyme assays, drug design, drug screening, vaccine development and should closely represent the pathogen. This may also be true for studies involving proteins of other pathogenic microorganisms that are obtained from *E. coli* host expression systems. To effectively uncover functional aspects of identified proteins, important parameters including sequence conservation, multimerization and PTMs must be carefully considered while attempting to utilize *E. coli* or *M. smegmatis* as expression hosts for *M.tb* proteins. In summary, our findings indicate that *M.tb* H37Ra has the advantage of being closely related to *M.tb* H37Rv, thereby precluding most challenges associated with heterologous host expression. An added advantage is that *M.tb* H37Ra is an attenuated strain not requiring stringent containment facilities. The disadvantages of the system include the expense in terms of media, electroporation and long duration for *M.tb H37Ra* expression (10 days) as compared to *E. coli* (30 h) or *M. smegmatis* (48 h). A BSL2 compliant laboratory with appropriate biosafety cabinet and trained manpower would also be necessary to utilize this method. Overall, the use of *M.tb H37Ra* coupled with the availability of newer vectors for expression [34] could increase the yield of recombinant protein while retaining the fidelity of protein functionality.

## Methods

### Strains

*M.tb* H37Ra and *M. smegmatis* mc^2^155 strains were obtained from MTCC, IMTECH, Chandigarh. Both strains were maintained in Middlebrook’s 7H9 medium or 7H10 supplemented with glycerol, OADC and Tween 80 at 37 °C. *E. coli* BL21DE3 (Novagen) was used for expression by pET vector.

### Plasmids and constructs

pET28c (Novagen) was utilized for expression in *E. coli.* The *E. coli*-*Mycobacterium* shuttle vector pSC300 was received as a gift from Prof. Y Av Gay (Department of Medicine, University of British Columbia). This vector contains the gene for enhanced GFP (Green Fluorescent Protein) downstream of a strong constitutive promoter superoxide dismutase (SOD) [35]. The GroEL/ES plasmid was received as a gift from Dr. T. Shrader [36] and Dr. JS Blanchard, Albert Einstein School of Medicine, USA [10].

### Cloning of *M.tb* GAPDH in pET28c

The *M.tb* GAPDH (Rv1436) gene sequences were retrieved from TBDB (Tuberculosis Data Bank, http://www.tbdb.org). PCR amplification was carried out using *M.tb* genomic DNA (BEI resources) to incorporate required restriction sites (Additional file 3: Table 1). Products were then cloned in the pET28c vector to obtain an inframe N-terminal His tag. The constructs were confirmed by sequencing and transformed into *E. coli* BL21DE3 cells, selection was done with kanamycin (30 µg/ml). This recombinant strain was also co-transformed along with the GroEL/ES plasmid [10, 36], where selection was with tetracycline (6 µg/ml) in addition to kanamycin. Individual colonies were analyzed for protein expression and purification which was carried out as described previously [10]. Cytosol, cell wall and inclusion body fractions were prepared by standard methods [37] and the presence of recombinant protein in various cellular fractions was determined by western blotting using monoclonal α-His antibody (1:3000) (Sigma).

### Cloning of *M.tb* GAPDH in pSC300 vector

The *M.tb* GAPDH gene was cloned in the pSC300 vector for expression in Mycobacterial strains. Splicing by overlap extension PCR (SOE PCR) was used to incorporate a thrombin cleavage site, a glycine linker at the C-terminal end of GAPDH, 8XHis tag and Cla I site in two sequential PCR reactions. Primers SOE 1 and 3 were used for the first reaction, the product was reamplified using primers SOE 2 and SOE 3 in the second step.

A similar approach was taken to create the N-terminal tagged GAPDH, SOE PCR was used to incorporate a enterokinase cleavage site and a glycine linker at the N-terminal end of GAPDH in the first PCR reaction. The primers SOE-N1 and SOE-N3 were used for this amplification step. The SOE product I was then amplified with SOE-N2 and SOE-N3 primers to include the 8XHis tag and BamHI site preceding the glycine linker (Additional file 3: Table 1). The parent pSC300 plasmid was digested with BamHI and ClaI enzymes to remove the existing GFP gene and the GAPDH-Thrombin site-8XHis (C terminal tagged fragment) or 8XHis-Enterokinase site-GAPDH (N terminal tagged fragment) fragment were inserted in the vector after digestion with the same enzymes. Plasmids pGAPDH-His and pHis-GAPDH were as confirmed by sequencing.

### Expression and localization of rGAPDH-His/rHis-GAPDH from *M. smegmatis and M.tb H37Ra*

Electroporation in *M. smegmatis* and *M.tb* H37Ra was done as described previously [9]. A single colony from each transformation was cultured in 7H9 media supplemented with OADC and 50 µg/ml hygromycin up to log phase. Sub cellular fractions were prepared and analysed for the presence of recombinant GAPDH by western blotting [9]. Briefly, 40 µg each of cytosol, cell membrane and cell wall fractions from *M. smegmatis* and *M.tb* H37Ra transformed with either plasmid were denatured by heating for 10 min at 95 °C and loaded on a 10 % SDS-PAGE. Control fractions of untransformed *M. smegmatis* and *M.tb* H37Ra were run alongside. Resolved proteins were either stained with coomassie blue or were transferred onto nitrocellulose membrane. Blots were probed with mouse monoclonal α-His (Sigma) for 1 h followed by incubation with goat α-mouse IgG-HRP (1:10,000 for 1 h), washed and developed with TMB/H_2_O_2_. Blots were also stripped and re-probed with 1:1000 dilution of rabbit polyclonal α-GAPDH followed by detection with 1:16,000 dilution of α-Rabbit-HRP (Sigma).

### Purification of rGAPDH-His and rHis-GAPDH from *M. smegmatis and M.tb H37Ra*

Start up cultures of *M. smegmatis* or *M.tb* H37Ra transformed with either plasmid were set up in 5 ml of 7H9 broth and utilized to inoculate a fresh 500 ml batch of media at an initial OD_600_ of 0.05. Cultures were then incubated at 37 °C with shaking at 200 rpm and maintained till log phase i.e. ~OD 0.8–1.0. Cells were harvested and washed once with PBS, pellets were then resuspended in cell lysis buffer (50 mM Tris pH 8.0, 150 mM NaCl) containing 1 mM PMSF and protease inhibitor cocktail (Sigma). Samples were sonicated and the lysate was centrifuged at 1000*g* for 10 min at 4 °C. The protein concentration of the lysate was adjusted to ~1.5 mg/ml and centrifuged (27,000*g*, 1 h at 4 °C) to pellet down the cell wall fraction. The supernatant (containing cytosol and cell membrane) was ultra centrifuged (1,00,000*g* for 2 h at 4 °C) to pellet down the cell membrane. The supernatant (cytosol) was filtered using a 0.2 µ filter and loaded onto a Ni–NTA column pre-equilibrated with 50 mM Tris pH 8.0 containing 150 mM NaCl. The column was thoroughly washed with buffer containing 30 mM imidazole followed by elution with buffer containing 250 mM imidazole. Proteins were immediately dialyzed into 50 mM Tris, 150 mM NaCl and 1 mM β-ME pH 8.0, aliquoted, flash frozen and stored at −80 °C for further use.

### GAPDH enzyme assay

GAPDH enzyme assays were performed essentially as described previously [9]. Briefly, 0.25 µg of purified enzyme was added into 200 µl of assay buffer (50 mM HEPES, 10 mM sodium arsenate and 5 mM EDTA, pH 8.5), 1 mM NAD^+^ and 2 mM glyceraldehyde-3-phosphate (G3P, Sigma) at 25 °C. Enzyme activity was measured as the increase in absorbance at 340 nm due to formation of NADH. To compare the activity of N- and C-terminally His tagged proteins, absorbance was monitored over 5 min. For negative controls, assays were carried out with buffer lacking the specific substrate G3P, these values were subtracted from the final absorbance.

### Determination of K_m_ and V_max_

For kinetic studies, Michaelis constant (K_m_) for G3P was determined by varying the concentration of G3P (from 0.02 to 2 mM) while maintaining a constant amount of NAD^+^ (1 mM). The K_m_ for NAD^+^ was determined by varying concentrations of NAD^+^ (from 0.01 to 2 mM) and holding G3P concentration constant at 2 mM. Sodium arsenate was used at a saturating concentration of 10 mM for all assays. All enzyme reactions were carried out at 25 °C in assay buffer. The velocities were calculated from the initial linear portion of the reaction and considering the molar extinction coefficient of NADH (6220 M cm^−1^) along with the total enzyme concentration. Values of the Michaelis constants (*K*_m_) and maximal velocity (V_max_) were calculated using Lineweaver–Burk plot. One unit of enzyme activity was defined as the amount of enzyme that catalyzes the formation of 1 μmol NADH/min under the conditions used.

### Circular dichroism (CD) spectroscopy

CD spectra of MT-GAPDH-H and EC-H-GAPDH were recorded using a Jasco J-815 CD spectropolarimeter. The enzyme was dialyzed in 20 mM phosphate buffer pH 7.4 containing 1 mM NAD^+^ and was adjusted to a concentration of 0.25 mg/ml. The CD spectrum was recorded from 200 to 250 nm at 25 °C using a cell of 0.1 cm path length. Buffer containing NAD^+^ was taken as blank and its values were subtracted from the corresponding spectrum. Experiments were repeated thrice and the overlay spectrum of molar ellipticity v/s wavelength (nm) of representative experiment is presented [38].

### pH stability

Purified MT-GAPDH-H was incubated in different buffers ranging from pH 5.5–11.5 for 30 min at 25 °C. The buffers used were 50 mM MES buffer for pH 5.5 and 6.5, 50 mM HEPES buffer for pH 6.5, 7, 7.5, 8 and 8.5; 50 mM Glycine-NaOH for pH 8.5, 9 and 9.5; CAPS buffer (50 mM) was used for pH 9.5, 10, 10.5 and 11.5. After incubation the enzyme activity was determined at 25 °C using assay buffer. The activity was measured for 2 min, data is plotted as percentage residual activity versus pH.

### Thermal denaturation

The purified MT-GAPDH and EC-H-GAPDH along with 1 mM NAD^+^ were incubated at a range of temperatures between 15 and 80 °C for 10 min and then maintained at 25 °C for 2 min. The enzyme activities were measured as before. Data is plotted as percent residual activity versus temperature. For MT-GAPDH-H a parallel set of experiments was also run in the absence of 1 mM NAD^+^. Average of three independent experiments is represented.

### Effect of H_2_O_2_ and metal ions

All assays were carried out essentially as described for characterization of GAPDH from other species. Purified MT-GAPDH-H (1 µM) was supplemented with either 250 µM H_2_O_2_ or with individual metal ions (Cu^+2^, Cd^+2^, Co^+2^ or Fe^+2^) at 4 °C for 10 min in 50 mM Tris–HCl buffer pH 7.4 with or without 1 mM 2β-ME. GAPDH enzyme activity was measured for 2 min in assay buffer lacking EDTA. Experiments were repeated thrice independently, data is plotted as % residual activity ±SD (n = 3).

### Far western blotting

Far western blotting was carried out as described earlier [9] briefly 3 µg of transferrin was run on a 10 % SDS-PAGE and transferred to nitrocellulose. The membrane was then probed with 10 µg/ml of either recombinant GAPDH from *E. coli* or *M. smegmatis* or *M.tb* H37Ra. Binding of GAPDH to transferrin was detected using α-GAPDH, all experiments were repeated thrice.

### Co-immunoprecipitation of transferrin and rGAPDH

Co-immunoprecipitation was performed essentially as described previously [9]. Briefly, 2.5 µM of transferrin-biotin was first mixed with 10 µl of streptavidin paramagnetic particles (SAPMP’s, Pierce) for 1 h at 4 °C on a rotospin. The particles were washed with PBS and incubated for 2 h at 4 °C with 2.5 µM of recombinant GAPDH purified from each of the host systems. After washing with PBS, particles were resuspended in 30 µl of Laemmli sample buffer, boiled and loaded on a 10 % SDS-PAGE gel. Captured GAPDH was detected by western blotting using α-GAPDH antibody as described previously. Captured transferrin was also detected using 1:1000 rabbit α-Tf antibody (Abcam), to serve as the loading control. Blots were analyzed by densitometry using Quantity One software (BioRad) to quantitate the amount of GAPDH captured. Experiments were repeated three times independently, data is plotted as % binding ±SD. Statistical analysis was done using student’s t test.

### Cloning of *M.tb* H37Rv PykA and Enolase in *E. coli* expression vectors

The *M.tb* PykA (Rv1617) and Enolase (Rv1023) genes were PCR amplified from *M.tb* H37Rv genomic DNA (BEI resources) with forward and reverse primers containing *Nde*I and *Hind*III sites respectively (Additional file 3: Table 1). The products were then individually cloned in the pET 30a (PykA, C-terminal His tagged) or pET28c (Enolase, N-terminal His tagged) vectors. Plasmids pET30-PykA-His and pET28-His-Eno were confirmed by sequencing.

### Cloning of *M.tb* H37Rv PykA and Enolase in mycobacterial expression vector

The *M.tbpykA* gene was cloned in the pSC300 vector using Splicing by overlap extension PCR (SOE PCR) to incorporate an enterokinase cleavage site and a glycine linker at the C-terminal end of PykA in the first PCR reaction. The primers PykA-SOE-1 and PykA-SOE-2 were used for this amplification step (Additional file 3: Table 1). The SOE product I was then amplified with PykA-SOE-1 and PykA-SOE-3 primers to include the 8XHis tag and ClaI site after the glycine linker. The parent pSC300 plasmid was digested with *Bam*HI and *Cla*I enzymes to remove the existing GFP gene and the PykA-Enterokinase site-8XHis fragment was inserted in the vector after digestion with the same enzymes.

For Enolase, the parent pSC300 plasmid was digested with *Bam*HI and *Cla*I enzymes to remove the existing GFP gene and a cassette (containing 8X His tag followed by *Nde*I and *Hind*III sites) was inserted in the vector. This cloned vector pHis300 was further digested with *Nde*I and *Hind*III and the same PCR amplified *M.tb* H37Rv enolase product was digested and inserted at this site to obtain in-frame N-terminal His tag gene. Both plasmid pPykA-His and pHis-Enolase were confirmed by sequencing. Plasmids were transformed into *M. smegmatis* or *M.tb* H37Ra strains as described previously for pGAPDH-His.

### Expression and localization of rPykA-His and rHis-Enolase in *E. coli*

The plasmids were transformed into *E. coli* BL21DE3 GroEL/ES strain, selection was done with kanamycin (30 µg/ml) and tetracycline (6 µg/ml). Single colonies were isolated and analyzed for protein expression and purification. Cultures were grown in LB media at 30 °C and induced with 0.1 mM IPTG at an OD_600_ of 0.5–0.6, cultures were then maintained at 18 °C for 15 h. Cells were harvested by centrifugation and pellets were stored at −20 °C. Cell pellets were resuspended in lysis buffer PykA (50 mM Tris pH 8.0, 150 NaCl) and Enolase (50 mM Tris pH 8.0) containing 1 mM PMSF and protease inhibitor cocktail. Cytosol, cell wall and inclusion body fractions were prepared by standard methods as described earlier for rGAPDH and the presence of recombinant protein in various cellular fractions was determined by western blotting using monoclonal α-His antibody (1:3000).

### Expression and localization of rPykA-His, rHis-Enolase from *M. smegmatis and M.tb H37Ra*

Electroporation and preparation of sub cellular fractions from *M. smegmatis* and *M.tb* H37Ra was done as described for GAPDH. Samples (40 µg each) were analyzed on a 10 % SDS-PAGE, control fractions of untransformed *M. smegmatis* and *M.tb* H37Ra were run alongside. Resolved proteins were either stained with coomassie blue or were transferred onto nitrocellulose membrane. Blots were probed with mouse monoclonal α-His (Sigma) for 1 h followed by incubation with goat α-mouse IgG-HRP (1:10,000 for 1 h), washed and developed with TMB/H_2_O_2_.

Proteins have been referred to as EC-PykA-His, MS-PykA-H, MT-PykA-H and EC-H-Enolase, MS-H-Enolase, MT-H-Enolase to reflect the expression host, position of tag and protein.

### Purification of recombinant PykA from *E. coli*, *M. smegmatis or M.tb H37Ra strains*

The induced cell pellet was resuspended in lysis buffer (50 mM Tris pH 8.0, 150 mM NaCl) containing PMSF and protease inhibitor cocktail. Cells were processed as described for recombinant GAPDH purification from *E. coli.*

Start up cultures and cell pellets from *M. smegmatis* or *M.tb* H37Ra transformed with pPykA-His were set up as described for GAPDH. Pellets were resuspended in lysis buffer (50 mM Tris, pH8.0, 150 mM NaCl) containing 1 mM PMSF and protease inhibitor cocktail (Sigma). Lysates from all three hosts were processed as before to obtain the cytosol, the pre-filtered fraction loaded onto a Ni–NTA column pre-equilbrated with lysis buffer. The column was thoroughly washed with buffer containing 35 mM imidazole followed by elution with buffer containing 250 mM imidazole. Proteins were immediately dialyzed into 50 mM HEPES pH 7.5 or 50 mM HEPES pH 7.5 supplemented with either 10 mM MgCl_2_ or 100 mM KCl. Protein was aliquoted, flash frozen and stored at −80 °C for further use. Purification was confirmed by coomassie staining of 10 % SDS-PAGE and by western blotting using monoclonal α-His antibody. PykA was also assessed using affinity purified Rabbit polyclonal antibody (0.5 µg/ml). The antibody was custom synthesized by Bioneeds, Bangalore, India using IB fraction obtained from *E. coli.* The ~50 kD protein that co-elutes with MS-PykA-H was subjected to PMF as done for GAPDH.

### Enzymatic assay for Pyruvate kinase

Pyruvate kinase enzyme assay was performed to compare the activity of recombinant protein purified from three different sources. To evaluate stability, samples were stored at 4 °C for a period of 0–72 h. Pyruvate kinase assay buffer [39] (100 µl) containing 50 mM HEPES pH 7.5, 10 mM MgCl_2_, 50 mM KCl, 2 mM adenosine diphosphate, 10 mM sodium phosphoenol pyruvate, 0.3 mM NADH, 1.5 U lactate dehydrogenase was preincubated at 25 °C for 3 min. Pyruvate kinase (100 ng) was added and the decrease in absorbance at 340 nm was monitored for 1 min. For negative controls, assays were carried out with buffer lacking the specific substrate sodium phosphoenol pyruvate; these values were subtracted from the final absorbance. Experiments repeated thrice in duplicates. For each set of experiments, data has been plotted as % residual activity ±SD v/s time (h) by considering activity of MT-PykA-H at 0 h as 100 %. Data was analyzed by Student’s unpaired t test.

### Purification of recombinant Enolase from *E. coli*, *M. smegmatis and M.tb H37Ra strains*

The induced *E. coli* cell pellet was resuspended in lysis buffer i.e. 50 mM Tris pH 8.0 containing PMSF and protease inhibitor cocktail. Cells were processed as described for recombinant GAPDH purification from *E. coli.* Start up cultures and cell pellets from *M. smegmatis* or *M.tb* H37Ra transformed with pHis-Eno were set up as described for GAPDH. Pellets were resuspended in lysis buffer (50 mM Tris pH 8.0 containing PMSF and protease inhibitor cocktail). Lysates from all three hosts were processed as described previously. The column was thoroughly washed with buffer containing 20 mM imidazole followed by elution with buffer containing 250 mM imidazole. Proteins were immediately dialyzed into 50 mM HEPES, pH 7.0, aliquoted, flash frozen and stored at −80 °C for further use. To ascertain the effect of Mg^2+^, purification was also performed by supplementing all buffers with 5 mM MgSO_4_. Purification was confirmed by coomassie staining of 10 % SDS-PAGE and western blotting using monoclonal α-His antibody.

### Enzyme assay for Enolase

Enolase enzyme assay was performed as described previously [40] to compare the activity of recombinant protein purified from three different hosts. The effect of Mg^2+^ on enzyme activity and enzyme stability over a period of 0–10 h was evaluated time points of 0, 6 and 10 h are depicted. Enolase assay buffer [50 mM HEPES pH 7.0, 7.7 mM KCl, 10 mM MgSO_4_ and 9 mM D (+)-2 phosphoglyceric acid sodium salt (2-PG)] was preincubated at 37 °C for 3 min. Purified enolase was added at a concentration of 1 µg per 100 µl of buffer at 37 °C, the increase in absorbance at 240 nm was monitored for 5 min. For negative controls, assays were carried out with buffer lacking the specific substrate 2-PG, these values were subtracted from the final absorbance. Experiments repeated thrice in duplicates, for each set of experiments, data has been plotted as % residual activity ±SD v/s time (h) by considering activity of MT-H-ENO at 0 h as 100 %. Data was analyzed by Student’s unpaired t test.

## Additional files

10.1186/s12934-016-0537-0Expression of rGAPDH in *E. coli*. **a.** Expression of EC-H-GAPDH in cellular fractions of *E. coli*-GroEL/ES strain assessed by 10 % SDS PAGE and **b.** confirmation by western blotting using Mouse α His antibody. **c.** Affinity purified EC-H-GAPDH and western blot with α-GAPDH and α-His to confirm purification from *E. coli* host cytosol. **d.** ClustalW analysis of GAPDH from *M.tb* H37Rv, *M.tb* H37Ra, *M. smegmatis* and *E. coli*.
10.1186/s12934-016-0537-0Intact mass analysis and isoelectric point of recombinant *M.tb* GAPDH. MALDI-TOF confirms the presence of a single protein corresponding to **a.** MT-GAPDH-H and **b.** EC-H-GAPDH. Two dimensional gel analysis of rGAPDH purified from different source **c.** MT-rGAPDH **d.** MS-rGAPDH **e.** EC-rGAPDH where the gel was either stained with coomassie or western blot using rabbit α-GAPDH or mouse α-His. **f.** Peptide mass fingerprinting (PMF) of ~ 50 kD *M. smegmatis* protein that co-elutes with rPykA. Identified sequences are indicated in bold face.
10.1186/s12934-016-0537-0 Details of primers used, experimental and theoretical molecular weights, pI values and details of post-translational modifications in GAPDH.
10.1186/s12934-016-0537-0 Methodology for Peptide Mass Fingerprinting, Sequence analysis, 2D gel electrophoresis and LC MS/MS of GAPDH.

## Abbreviations

*M.tb*: *Mycobacterium tuberculosis*
GAPDH: glyceraldehyde-3-phosphate dehydrogenase
rGAPDH: recombinant GAPDH
PykA: pyruvate kinase
Eno: Enolase
G3P: glyceraldehyde-3-phosphate
NAD^+^: nicotinamide adenine dinucleotide
Tf: transferrin
PTMs: post-translational modifications
PMF: peptide mass fingerprinting
