# Supplementary figures and images for: Mycobacterium tuberculosis H37Ra: a surrogate for the expression of conserved, multimeric proteins of M.tb H37Rv
Source: Microb Cell Fact. 2016 Aug 11;15:140. doi: 10.1186/s12934-016-0537-0 (PMC4982137; doi:10.1186/s12934-016-0537-0)

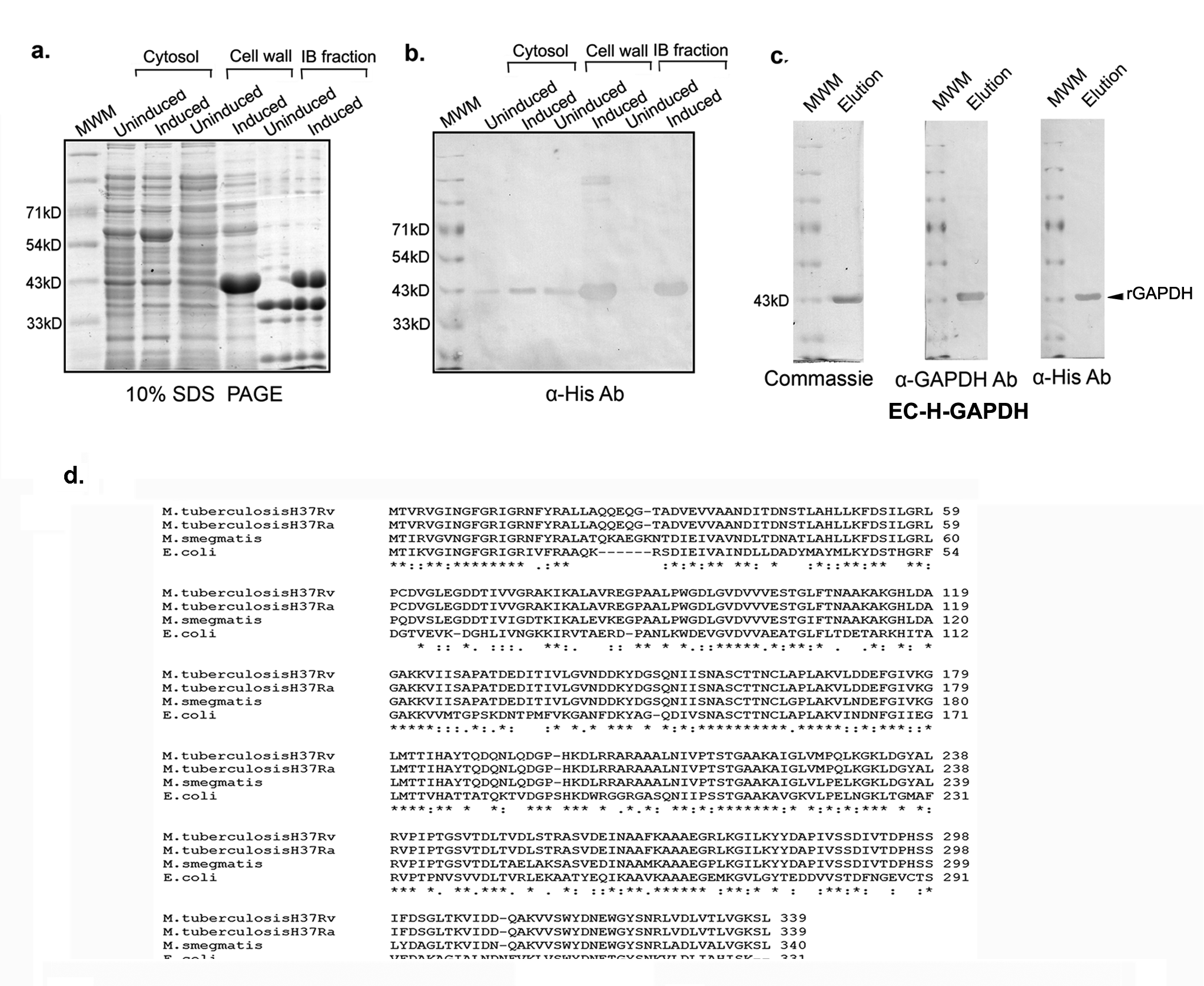

Supplement: Supplementary file 1 — 10.1186/s12934-016-0537-0Expression of rGAPDH in E. coli. a. Expression of EC-H-GAPDH in cellular fractions of E. coli-GroEL/ES strain assessed by 10 % SDS PAGE and b. confirmation by western blotting using Mouse α His antibody. c. Affinity purified EC-H-GAPDH and western blot with α-GAPDH and α-His to confirm purification from E. coli host cytosol. d. ClustalW analysis of GAPDH from M.tb H37Rv, M.tb H37Ra, M. smegmatis and E. coli. [file 12934_2016_537_MOESM1_ESM.tif]

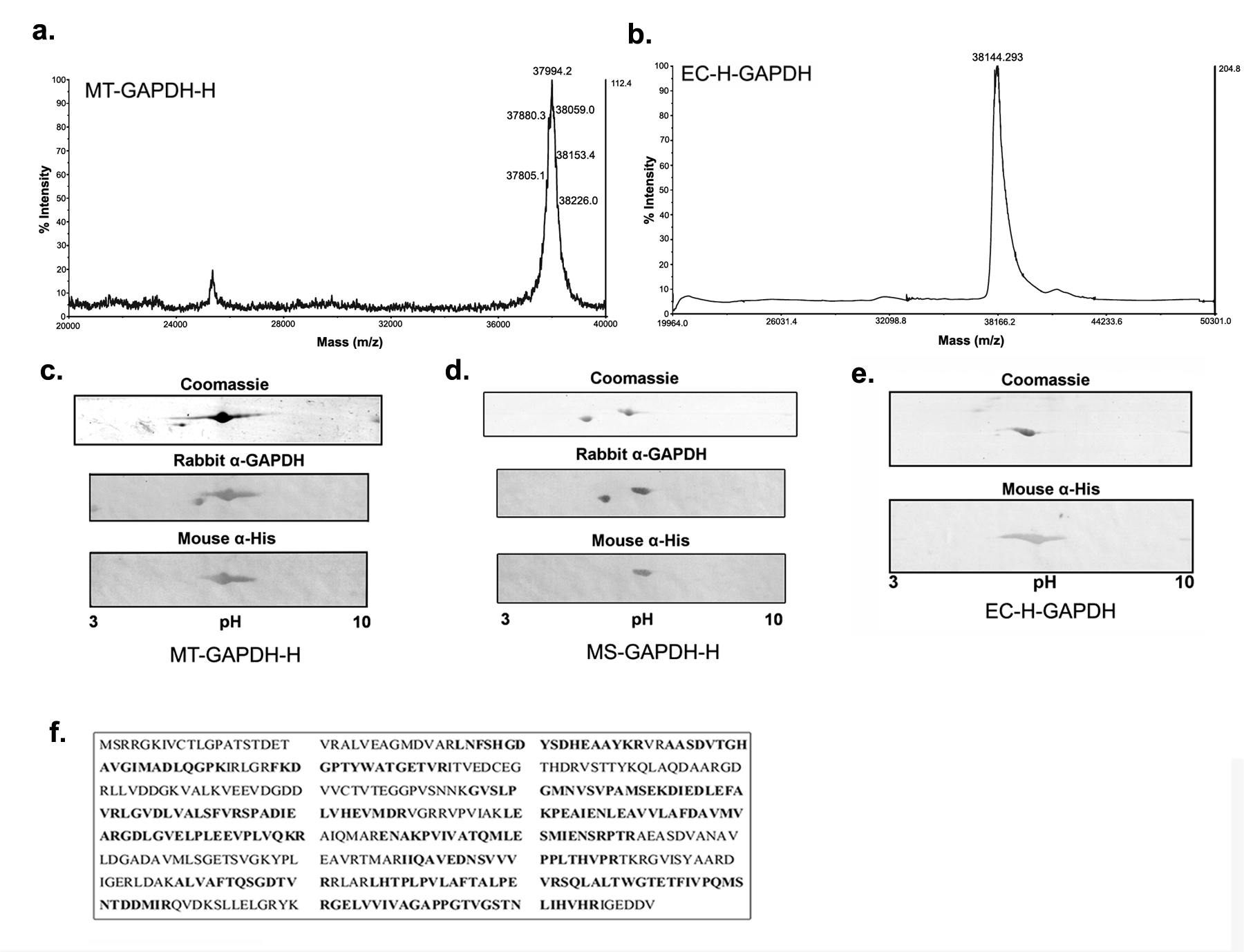

Supplement: Supplementary file 2 — 10.1186/s12934-016-0537-0Intact mass analysis and isoelectric point of recombinant M.tb GAPDH. MALDI-TOF confirms the presence of a single protein corresponding to a. MT-GAPDH-H and b. EC-H-GAPDH. Two dimensional gel analysis of rGAPDH purified from different source c. MT-rGAPDH d. MS-rGAPDH e. EC-rGAPDH where the gel was either stained with coomassie or western blot using rabbit α-GAPDH or mouse α-His. f. Peptide mass fingerprinting (PMF) of ~ 50 kD M. smegmatis protein that co-elutes with rPykA. Identified sequences are indicated in bold face. [file 12934_2016_537_MOESM2_ESM.tif]
