# Supplementary material for: Mycobacterium tuberculosis H37Ra: a surrogate for the expression of conserved, multimeric proteins of M.tb H37Rv
Source: Microb Cell Fact. 2016 Aug 11;15:140. doi: 10.1186/s12934-016-0537-0 (PMC4982137; doi:10.1186/s12934-016-0537-0)
Supplement: Supplementary file 3 — 10.1186/s12934-016-0537-0 Details of primers used, experimental and theoretical molecular weights, pI values and details of post-translational modifications in GAPDH. [file 12934_2016_537_MOESM3_ESM.doc]

**Additional File 3**

Table 1: Details of constructs, primers and PCR conditions

| **S.no** | **Construct** | **Primers** | **PCR conditions** |
| --- | --- | --- | --- |
| 1. | p*M.tb* His-GAPDH  (with N terminal His tag in pET28c) | **Forward:**  5’CTTACATCACATATGACGGTCCGAGTAGGC  **Reverse:**  5’CTTATTATGGATCCCTAGAGCGACTTGCCGAC | 93°C, 3min; 93°C, 40 sec; 57.5°C, 1 min 72°C, 1:20 min (5 cycles); 93°C, 40 sec; 69°C, 1 min 72°C, 1:20 min (30 cycles); 72°C for 10 min. |
| 2. | pGAPDH-8XHis with C terminal His tag in pSC300 vector | **SOE 1 (Forward):** 5’GTACTCACAGGATCCATGACGGTCCGAGTAGG  **SOE 2 (Reverse):** 5’TGGTGTCCTCCTCCTCCCGATCCGCGTGGCACGAGTGACTTTCCGACCAG  **SOE 3 (Reverse):** 5’GTTATTCAATCGATCTAGTGGTGGTGGTGGTGGTGGTGGTGTCCTCCTCCTCC | SOE PCR I: 93°C, 3min; 93°C, 40 sec; 55°C, 1 min 72°C, 1:20 min (5 cycles); 93°C, 40 sec; 68°C, 1 min 72°C, 1:20 min (30 cycles); 72°C for 10 min.  SOE PCR II: 93°C, 3min; 93°C, 40 sec; 58°C, 1 min 72°C, 1:20 min (5 cycles); 93°C, 40 sec; 68°C, 1 min 72°C, 1:20 min (30 cycles); 72°C for 10 min. |
| 3. | pHis-GAPDH with N-terminal 8X His tag in pSC300 vector | **SOE N1 (Forward):**  5’CATCACGGTGGTGGTGGAGACGACGACGACAAAATGACGGTCCGAGTAGG  **SOE N2 (Forward):** 5’GTTTAAACGGATCCATGCATCATCATCATCATCATCATCACGGTGGTGGTGG  **SOE N3 (Reverse):**  5’GTTATTCCATCGATCTAGAGAGACTTGCCGACC | SOE PCR I: 93°C, 3min; 93°C, 40 sec; 55°C, 1 min 72°C, 1:20 min (5 cycles); 93°C, 40 sec; 68°C, 1 min 72°C, 1:20 min (30 cycles); 72°C for 10 min.  SOE PCR II: 93°C, 3min; 93°C, 40 sec; 60°C, 1 min 72°C, 1:20 min (5 cycles); 93°C, 40 sec; 68°C, 1 min 72°C, 1:20 min (30 cycles); 72°C for 10 min. |
| 4. | pET30a-PykA-His (with C terminal 6X tag in pET30a) | **Forward:**  5’CAGTAAAACATATGACGAGACGCGGG  **Reverse:**  5’GTTATATCAAGCTTCACGTCATCTTCCCCG | 93°C, 3min; 93°C, 40 sec; 54°C, 1 min 72°C, 1:50 min (5 cycles); 93°C, 40 sec; 66°C, 1 min 72°C, 1:50 min (30 cycles); 72°C for 10 min. |
| 5. | pPykA-His with C terminal 8X His tag in pSC300 vector | **PykA-SOE-1 (Forward):**  5’ GAAATACAAGGATCCATGACGAGACGCGGG  **PykA-SOE-2 (Reverse):**  5’GTGGTGTCCTCCTCCTCCGCTCTTATCATCATCATCGACGTCATCTTCCCC  **SOE 3 (Reverse):** 5’GTTATTCAATCGATCTAGTGGTGGTGGTGGTGGTGGTGGTGTCCTCCTCCTCC | SOE PCR I: 93°C, 3min; 93°C, 40 sec; 50°C, 1 min 72°C, 1:50 min (5 cycles); 93°C, 40 sec; 62°C, 1 min 72°C, 1:50 min (30 cycles); 72°C for 10 min.  SOE PCR II: 93°C, 3min; 93°C, 40 sec; 57°C, 1 min 72°C, 1:50 min (5 cycles); 93°C, 40 sec; 66°C, 1 min 72°C, 1:50 min (30 cycles); 72°C for 10 min. |
| 6. | pET28c-His-Enolase (with N terminal 6X His tag in pET28a).  The PCR product was also used for cloning in *E.coli-M.tb* shuttle vector pHis300. | **Forward:**  5’- GAAAAAAACATATGCCGATTATCGAGC  **Reverse:**  5’- GTTTACACAAGCTTCTATTTCGTCTCGCATGC | 93°C, 3min; 93°C, 40 sec; 52°C, 1 min 72°C, 1:30 min (5 cycles); 93°C, 40 sec; 65°C, 1 min 72°C, 1:30 min (30 cycles); 72°C for 10 min. |

Table 2: Comparison of experimental and theoretical molecular weight of rGAPDH

| **S.no** | **Samples** | **Experimental Mass (Da)** | **Expected Mass (Da)** | **Mass Difference (Da)** |
| --- | --- | --- | --- | --- |
| 1. | MT-GAPDH-H | 37994.2 | 37782 | 212.2 |
| 2. | EC-H-GAPDH | 38144.3 | 38124 | 20.3 |

Table 3: Comparison of experimental and theoretical isoelectric point (pI) values of *M.tb* GAPDH

| **S.no** | **Samples** | **Theoretical pI** | **Experimental pI** |
| --- | --- | --- | --- |
| 1. | Native *M.tb* H37Ra GAPDH | 5.19 | 5.6 |
| 2. | MT-rGAPDH | 5.93 | 6.43 |
| 3. | Native *M.smegmatis* GAPDH | 5.12 | 5.42 |
| 4. | MS-rGAPDH | 5.93 | 6.37 |
| 5. | EC-rGAPDH | 5.93 | 6.57 |

**Table 4**: Post translational modifications in rGAPDH from three different hosts

|  | **EC-H-GAPDH** | **MS-GAPDH-H** | **MT-GAPDH-H** | **Native *M.smegmatis* GAPDH** |
| --- | --- | --- | --- | --- |
| MOWSE score | 2813 | 5200 | 4373 | 2067 |
| Sequence coverage | 92% | 94% | 92% | 93% |
| **Modifications** |  |  |  |  |
| Deamidation | 13 | 18 | 14 | 15 |
| Phosphorylation (ST) | 3 | 9 | **6** | 3 |
| Oxidation | 3 | 3 | 3 | 2 |
| Hexose | -- | 1 | -- | 1 |
| Glycosyl | -- | 1 | -- | -- |
| Methylation | 8 | 14 | **17** | 8 |
| Dimethylation | 4 | 6 | 3 | 0 |
| Pyro-glu | 2 | 2 | 2 | 2 |
| Pro-pyro-glu | 3 | 7 | 4 | 0 |
| Acetylation | 3 | 4 | 3 | 1 |
| Nitrosyl | 1 | 1 | 1 | - |
| Propionamide | 1 | 3 | 1 | 2 |
| Succinyl | 0 | 0 | **1** | 1 |
| **Total** | **41** | **69** | **55** | **36** |

Other modifications including myristoylation, farnesylation, GPI anchor, palmitoylation, ADP ribosylation were not detected in the analyzed samples
