# Supplementary material for: Mycobacterium tuberculosis H37Ra: a surrogate for the expression of conserved, multimeric proteins of M.tb H37Rv
Source: Microb Cell Fact. 2016 Aug 11;15:140. doi: 10.1186/s12934-016-0537-0 (PMC4982137; doi:10.1186/s12934-016-0537-0)
Supplement: Supplementary file 4 — 10.1186/s12934-016-0537-0 Methodology for Peptide Mass Fingerprinting, Sequence analysis, 2D gel electrophoresis and LC MS/MS of GAPDH. [file 12934_2016_537_MOESM4_ESM.doc]

***Additional File 4:***

***Mycobacterium tuberculosis* H37Ra - a surrogate for the expression of conserved, multimeric proteins of *M.tb* H37Rv.**

Vishant Mahendra Boradia^1^, Pravinkumar Patil^1^, Anushri Agnihotri^1^, Ajay Kumar^1^, Kalpesh Kumar Rajwadi^1^, Ankit Sahu^1^, Naveen Bhagath^1^, Navdeep Sheokand^2^, Manoj Kumar^2^, Himanshu Malhotra^2^, Rachita Patkar^1^, Navi Hasan^1^, Manoj Raje^2^ & Chaaya Iyengar Raje^1^, *

From:  ^1^. National Institute of Pharmaceutical Education and Research (NIPER), Phase X, Sector 67, SAS Nagar, Punjab- 160062, INDIA.

^2^. Council of Scientific and Industrial Research-Institute of Microbial Technology (CSIR-IMTECH), Sector 39 A, Chandigarh 160036, INDIA

**Supplementary Methods**

**Peptide Mass Fingerprinting (PMF) and Intact Mass Analysis** Intact mass of rGAPDH purified from *M.tb* H37Ra was analyzed using MALDI-TOF at Vimta Labs Hyderabad, India. The ~40 kD band observed to co-elute with rGAPDH on purification from *M.smegmatis* was excised from 10% SDS-PAGE stained gels and also subjected to peptide mass fingerprinting. Database searching (Swiss-Prot) was restricted to the *Mycobacterium tuberculosis* complex, and protein identification were performed with MASCOT Software (http://www.matrixscience.com), with: trypsin plus one missed cleavage, carboxyamidemethylation as a fixed modification, methionine oxidation as a variable modification and a mass tolerance of 0.5 Da for the precursor molecular weight. The criteria to accept a protein hit as a valid identification was multiple tryptic peptide matches to the protein sequence and a significance of *p*<0.05 [1].

**Sequence analysis** Sequences of; *M.tuberculosis* H37Ra (YP_001282750.1), *M.tb* H37Rv (CAB09248.1)*,* *M.smegmatis* (YP_887400.1), and *E.coli*. (AP_002398.1) were retrieved from National Center Biotechnology Information (NCBI) protein database. Amino acid sequences were aligned using ClustalW2 software from the European Bioinformatics Institute (<http://www.ebi.ac.uk/Tools/msa/clustalw2/>).

**2D Gel Electrophoresis** The purified MT-GAPDH-H (5µg) was treated with ReadyPrep™ 2-D-cleanup kit (Biorad). Protein pellet was solubilized in 125µl of rehydration buffer [7 M urea, 2 M thiourea, 3% 3[3-cholaminopropyl diethylammonio]-1-propane sulfonate (CHAPS), 50 mM DTT and 0.5% v/v of Biolytes pH 3–10], and the sample was applied onto a linear pH 3–10 ReadyStrip™ IPG strip (7 cm, Biorad). Isoelectric focusing was performed on a Bio-Rad PROTEAN® IEF cell at 20°C and current maintained at 50µA/strip using the following program: (1) passive rehydration for 12 h; (2) 250 V for 2 hr (linear voltage ramping); (3) 250 V for 1 hr (rapid voltage ramping); (4) 3000 V for 4 hr (linear voltage ramping) and (5) 10,000 V for 6 hr (linear voltage ramping). After electrofocusing, the strip was reduced for 15 min in equilibration buffer (6 M urea, 0.375 M Tris pH 8.8, 2% sodium dodecyl sulfate (SDS), 20% glycerol) containing 1% DTT and then alkylated for 15 min in equilibration buffer containing 1.25% Iodoacetamide [2]. Strips were then overlaid onto 12.5% SDS-PAGE and the second dimension run was performed. Proteins from SDS–PAGE were stained with Coomassie blue or transferred onto a nitrocellulose membrane for western blotting. Blots were probed with mouse α-His antibody or rabbit polyclonal α-GAPDH antibody.

**LC-MS/MS analysis of GAPDH** Purified *M.tb* GAPDH from all three host strains was individually run on SDS PAGE and stained with coomassie brilliant blue. Bands corresponding to *M.tb* GAPDH were excised and destained with 200 mM of NH_4_HCO_3_ in 40% acetonitrile (ACN) at 37°C for 30 min. Gel pieces were dried in a Speed Vac® and incubated at 37°C in digestion buffer containing 20 ng/µl trypsin (Sigma) alongwith 40 mM of NH_4_HCO_3_ in 9% acetonitrile for 18 hr. Finally, supernatant was collected for digested peptides and subjected to LC-MS/MS analysis. Peptides were analyzed by ultra-high performance liquid chromatography (UPLC)/ESI/MS/MS with mass spectrometer (Q-TOF 6550, Agilent). Peptides were separated using a C_4_ reversed phase 3.5 µm, 2.1x150 mm analytical column (X Bridge™ BEH 300, Waters), Sample (8 µl) was injected and flow rate was maintained at 400 µl/min. The mobile phase consisted of 90% solution A (0.1% formic acid, 90% water,10% ACN) and 10% of solution B (0.1% formic acid, 90% ACN, 10% water) for 15 min , 70% solution A: 30% solution B for 8 min, 40% solution A : 60% solution B for 5 min, 10% solution A : 90% solution B for 2 min, 50% solution A : 50% solution B for 2 min and 90% solution A :10% solution B for 2 min over a time period of 32 min. The capillary voltage was 1.5 kV and dry gas flow rate of 13 l/min was used with temperature of 220°C. The scan range used was 100-3200 m/z. Data analysis was performed essentially as described earlier [3]. Protein identification was performed by searching in National Center for Biotechnology Information non redundant database (NCBInR) using Mascot program (Matrix Science) with the following parameters: peptide mass tolerance, 1.2 Da; MS/MS ion tolerance, 0.6Da; taxonomy was limited to *Mycobacteria tuberculosis* complex; up to 1 missed trypsin cleavage site allowed; the variable modifications considered were: acetylation (K, Nterm), deamidation (N,Q), methylation (D,E), dimethylation (N,R,K), oxidation (M,C), phosphorylation (S,T,Y), propionamide (C), Pro->Pyro-Glu (P), Pyro-Glu (N-term, E,Q), nitrosylation (C), succinylation (K), Hexose (N), Glycosyl(P), ADP-ribosylation (C), Palmitoylation (C), Myristoylation (N term G), Farnesyltion (C), GPI Anchor (Protein C term). Only significant hits as defined by MASCOT probability analysis were considered. In addition, a minimum total score of 50 comprising at least a peptide match of ion score more than 20 were arbitrarily set as threshold for acceptance [3].

**References:**

1. Cottrell JS, London U. Probability-based protein identification by searching sequence databases using mass spectrometry data. Electrophoresis. 1999;20(18):3551-67.

2. Sheokand N, Malhotra H, Kumar S, Tillu VA, Chauhan AS, Raje CI *et al*. Moonlighting cell surface GAPDH recruits Apo Transferrin to effect iron egress from mammalian cells. J Cell Sci. 2014;127:4279-91.

3. Seo J, Jeong J, Kim YM, Hwang N, Paek E, Lee K-J. Strategy for comprehensive identification of post-translational modifications in cellular proteins, including low abundant modifications: application to glyceraldehyde-3-phosphate dehydrogenase. J Proteome Res. 2008;7(2):587-602.
